# Design and Analysis of the Bionic Mechanical Foot with High Trafficability on Sand

**DOI:** 10.1155/2020/3489142

**Published:** 2020-07-11

**Authors:** Rui Zhang, Hao Pang, Haijin Wan, Dianlei Han, Guoyu Li, Lige Wen

**Affiliations:** ^1^Key Laboratory of Bionic Engineering, Ministry of Education, Jilin University, Changchun, China; ^2^School of Mechanical and Aerospace Engineering, Jilin University, China

## Abstract

The ostrich foot has excellent travelling performance on sand and plays a vital role in efficient locomotion. The tendon-bone assembly characteristics of an ostrich foot were studied by gross anatomy, and the 3D model of ostrich foot was reconstructed and analyzed using reverse engineering techniques. Further, the bionic mechanical foot, suitable for locomotion on loose sand, was designed based on the structural characteristics of ostrich foot and its rigid-flexible coupling mechanism of tendon-bone synergies. The travelling performance on sand of the bionic mechanical foot was tested on a test platform by using Simi Motion. After analyzing the angle changes of the ankle joint and the metatarsophalangeal (MTP) joint, the displacement changes of the knee joint, the ankle joint, the MTP joint, and each phalanx along the *z*-axis, the plantar pressure distribution, and the footprints, we drew the conclusion that the bionic mechanical foot is helpful to reduce the sinkage and improve the trafficability on sand ground. This study provides a new research method for the walking mechanism of a robot and deep space exploration platform walking on soft ground.

## 1. Introduction

Bionic walking robot has been widely applied in many fields due to its strong adaptability in complex environments, such as desert, mud, and ruins [1–3]. In the past decades, robot researchers have developed a series of bionic walking robots. The bipedal humanoid robot can imitate human appearance and behavior, and it is closer to human life. With the continuous development of science and technology, a series of significant progress has been achieved in the research of bipedal humanoid robots [4–6]. ASIMO2011 robot developed by HONDA [7], PETMAN robot and Atlas robot developed by Boston Dynamics [8, 9] are all typical representatives of humanoid robots, with good balance performance, which can simulate human jumping, squatting, and sitting. In recent years, many researchers have also invested in multilegged bionic robots, including quadruped robots and hexapod robots. For example, Boston Dynamics has made great progress in quadruped bionic robot, and has successively developed BigDog, LS3, WildCat, Spot, and SpotMini [10–13]. Besides, Cheetah, HyQ, and ASLP also promoted the development of quadruped bionic robots [14–18]. Osaka University designed the “Asterisk” hexapod robot with spider as a bionic prototype [19], and NASA designed the “LEMUR” hexapod bionic robot with crab as a bionic prototype [20]. These robots are typical representatives of bionic hexapod robots. These bipedal and multilegged robots have their own superior performance; however, the feet of most existing bionic robots are integral structures, such as rectangular, semicylindrical, or spherical (oval) shape, supported by a damping or spring device. For example, the feet of ASIMO2011 are approximately elliptic, while the feet of Atlas are rectangular. These foot structures allow robots to walk steadily on the regular ground, but there have been few studies of mechanical foot suitable for soft terrains such as deserts, the surface of the moon, and Mars. Most robots adapt to a complex terrain by adjusting the postures of their feet, which remain in rigid contact with the ground. Under this background, the walking effect of robots on the soft ground is poor.

The hindlimb of the African ostrich (*Struthio camelus*) has a long-lasting, high-speed running ability. Taking the ostrich hindlimb as a bionic prototype and applying its superior performance to develop superior robots is an effective way. FastRunner, a bipedal robot designed with the ostrich hindlimb as the bionic prototype, can imitate the gait of the ostrich to achieve fast and efficient running, but its foot was simplified into a single toe structure containing three phalanges [21]. Cassie, an ostrich-like bipedal robot, is passively controlled with two rectangle feet, and can walk steadily on uneven roads [22, 23]. Although some robots were designed based on the characteristics of the ostrich hindlimb structure, they mainly focused on the design of the leg structure. Most of the studies targeted at speed, bearing capacity, and stability but did not consider the effect of the foot structure on locomotion of loose sand. The existing foot structures of these ostrich-like robots are too simplified due to the lack of analysis and application of biological locomotion mechanisms. Therefore, it is difficult to meet the application requirements of a robot on soft ground because of its weak sand fixation and flow limiting capacity. Therefore, the walking mechanism of a robot needs to be further studied and optimized, especially on soft ground.

Each phalanx touches the ground at the same time and leaves the ground in order when an ostrich walk on loose sand, which improves the travelling performance and trafficability on sand [24–26]. Gangl et al. dissected the hindlimb of an ostrich and gave the skeletal-tendon-ligament structure [27]. Smith et al. observed that the extensor tendon of the intertarsal joint of the ostrich can generate great force, which is suitable for energy storage [28]. Schaller et al. studied the toe function of the ostrich foot and found that the two-toed structure of the ostrich foot played a role in reducing pressure on the soft ground. In addition, the toenail is beneficial to improve the traction during ostrich locomotion [29]. These researches provide an important theoretical basis for the design of a bionic mechanical foot. We further studied the assembly characteristics of the tendon bone of the ostrich foot. Furthermore, imitating the rigid-flexible coupling mechanism of the tendon-bone synergies, we designed a bionic mechanical foot suitable for walking on loose sand.

## 2. Design and Fabrication of the Bionic Mechanical Foot

### 2.1. Analyses in Structure and Function of Ostrich Foot

**Figure 1:**
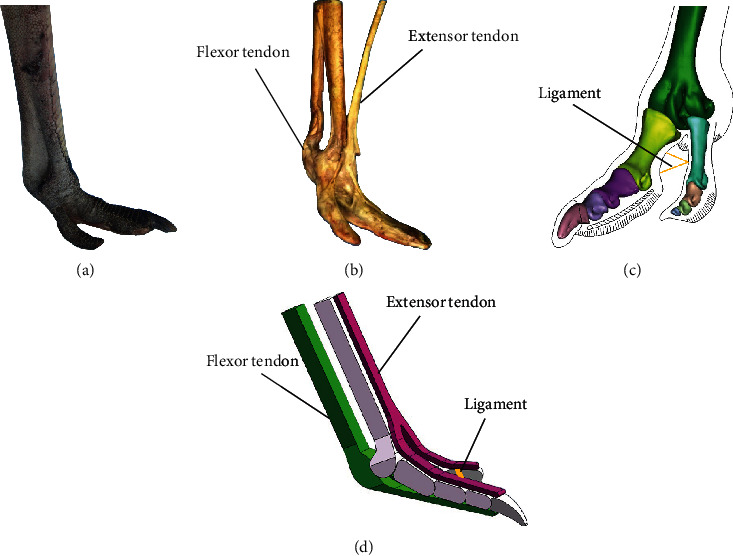
Ostrich foot specimen and 3D model. (a) Ostrich foot specimen. (b) Dissected ostrich foot. (c) 3D model of ostrich foot. (d) 3D simplified model of ostrich foot.

Ostrich (Struthio camelus Linnaeus, 1758) foot was purchased from an ostrich farm in Heilongjiang province (Figure 1(a)). Ethical approval was given by the Animal Experimental Ethical Inspection, Jilin University (reference No. 3130089). In order to study the excellent structural properties of ostrich foot and then apply it to the bionic design, we dissected the ostrich foot (Figure 1(b)). It was found that there were multiple tendons attached to the tarsometatarsus of the ostrich by dissection. Hutchinson et al. demonstrated that the flexor digitorum longus tendon serves as a “spring” during the stance phase, and the extensor digitorum longus tendon serves as a support during the lift-off phase [30, 31]. The interphalangeal ligament of the ostrich foot controls the opening and closing of the two toes.

The ostrich foot specimen was scanned using computed tomography (CT; Philips MX 8000 IDT 16 scanner, 220 kV, 220 mAs, and 1.25 mm slice thickness), and the three-dimensional (3D) model of the ostrich foot was reconstructed by Mimics and Geomagic Studio (Figure 1(c)). The bones of the ostrich foot include the tarsometatarsus, the third toe bone, and the fourth toe bone. The third toe bone includes the first phalanx, the second phalanx, the third phalanx, the fourth phalanx, and the toenail. In order to facilitate normalization, tendons with the same function and similar distribution position were first integrated into a whole. The tendons in the foot were classified and merged to produce two major tendons, the flexor tendon and extensor tendon. The 3D model of the ostrich foot was then simplified. The fourth phalanx of the main toe is small and closely related to the toenail, so they were integrated as a whole. Similarly, in the fourth toe bone, except for the larger size of the first phalanx, the other phalange sizes are smaller, so they were integrated into a whole. At the same time, with reference to the true morphological structure and positional relationship of the bones and tendons of the ostrich foot, a 3D simplified model of the ostrich foot was established (Figure 1(d)).

### 2.2. Structural Composition and Motion Principle

The bionic mechanical foot has a similar structure and function compared with the real ostrich foot. (1) In terms of structure, the bionic mechanical foot also has a two-toed structure, and an imitated ligament structure was added between the two toes. Moreover, in order to imitate the function of the flexor tendon and extensor tendon of the ostrich foot, the energy storage and shock absorption mechanism and the flexible passive control mechanism were designed. (2) In terms of function, the bionic mechanical foot can imitate the movement postures of the ostrich foot. During locomotion, its two toes can open and close, the phalanges of the third toe can leave the ground in order, and the toenails can thrust against the ground.

**Figure 2:**
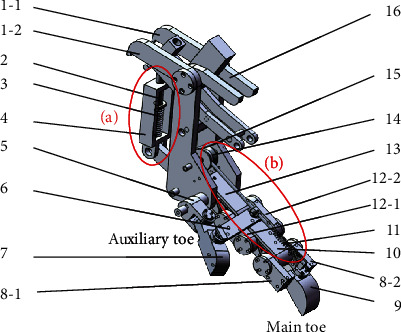
Bionic mechanical foot structure: (1) support plate, (2) stud, (3) flexion spring, (4) flexion frame, (5) the first phalanx of the auxiliary toe, (6) imitated ligament structure, (7) the second phalanx of the auxiliary toe, (8) the fourth phalanx of the main toe, (9) toenail structure, (10) the third phalanx of the main toe, (11) extension spring, (12) the second phalanx of the main toe, (13) the first phalanx of the main toe, (14) pulley, (15) wire rope, and (16) lever.

The structure of the bionic mechanical foot (Figure 2) mainly included the main structure, main toe, auxiliary toe, energy storage and shock absorption mechanism, and flexible passive control mechanism. The main toe and auxiliary toe imitated the third toe and the fourth toe of the ostrich foot, respectively, and the main structure was mainly composed of two supporting plates (1-1, 1-2). The main toe was mainly composed of the first phalanx (13), the second phalanx (12-1, 12-2), the third phalanx (10), and the fourth phalanx (8-1, 8-2). Each phalangeal joint was installed with a torsion spring, which plays the role of energy storage and shock absorption. The auxiliary toe was mainly composed of the first phalanx (5) and the second phalanx (7). The main toe and the auxiliary toe were connected on the same axis by a key, so that the two toes remained in the same plane during locomotion. The energy storage and shock absorption mechanism and the flexible passive control mechanism imitated the function of the tendon in the ostrich foot. The energy storage and shock absorption mechanism (a) were mainly composed of a flexion frame (4), a stud (2), and a flexion spring (3). The flexible passive control mechanism (b) was mainly composed of an extension spring (11), a wire rope (15), and a pulley (14).

The left end of the lever was connected with the energy storage and shock absorption mechanism and the linear servoelectric cylinder, and the right end was connected with the flexible passive control system. When the linear electric cylinder moved downward, the flexion spring in the energy storage and shock absorption mechanism was compressed to store energy and shock absorption. The extension spring in the flexible passive control mechanism was stretched to store energy, and the steel wire rope drove the imitated ligament structure to move to the left. Then, the auxiliary toe and the main toe separated to realize the opening of the two toes. After the lever moved to contact with the baffle, it continued to move upward, and the mechanical foot lifted. Finally, when linear electric cylinder moved upward, the flexion spring in the energy storage and shock absorption mechanism and the extension spring in flexible passive control mechanism released energy simultaneously, and the wire rope moved to the right to achieve the closure of two toes. When the lever moved upward, it touched the main structure and drove the whole foot to move. The running speed and running time of the linear servoelectric cylinder were adjusted by the controller.

### 2.3. Fabrication and Assembly

The actuator of the bionic mechanical foot was a linear servoelectric cylinder with a maximum load of 350 kg. The linear servoelectric cylinder was composed of a servomotor, electric cylinder, and connecting parts. The rated speed of the servomotor (ECMA C20604RS) was 3000 r/min and the rated torque was 1.27 N m.

**Figure 3:**
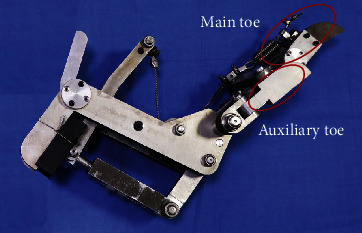
Bionic mechanical foot after fabrication and assembly.

In order to reduce the weight of the bionic mechanical foot, all parts were made of aluminum alloy while ensuring its rigidity. Because most of the parts on the mechanical foot were not standard parts, the processing method adopted was mainly linear cutting. The diameter of the wire rope adopted by the flexible passive control mechanism was 3 mm, the minimum breaking tension was 5.07 kN, and the maximum bearing capacity was 517.3 kg. The bionic mechanical foot was equipped with a variety of elastic elements; according to the function and type of elastic element, the flexion spring was used to replace the flexor tendon of ostrich foot, and the extension spring was used to replace the extensor tendon of ostrich foot. The bionic mechanical foot after assembly is shown in Figure 3, and its dimension parameters are shown in Table 1.

## 3. Test of Travelling Performance on the Sand

This section mainly introduces the process of the travelling performance test of the bionic mechanical foot on the test platform. The test was carried out on the solid ground and the loose sand at low speed, medium speed, and high speed, respectively, and the kinematics data of the bionic mechanical foot under six working conditions were processed by Simi Motion. Furthermore, the plantar pressure changes under six working conditions were measured by the thin-film pressure sensor. Combined with the kinematic analysis results of the bionic mechanical foot, the excellent travelling performance on sand of the bionic mechanical foot was verified.

### 3.1. Selection of Marker and Camera Position

**Figure 4:**
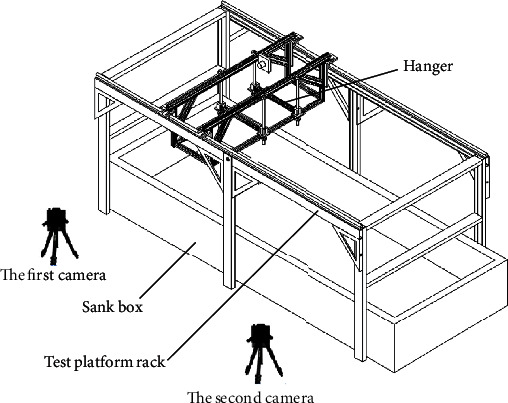
Test platform and camera position distribution diagram.

The bionic mechanical foot test platform was an important equipment for the travelling performance test of the bionic mechanical foot on sand and was mainly composed of a frame and a hanger. The overall dimensions of the frame were 4000 mm × 1750 mm × 1750 mm. Two 4 m long smooth rails were installed on the upper of the frame, and the hangers can move parallel along the rails in the sagittal plane. The main motor control system and the plantar pressure collection system were placed at the upper end of the hanger. The bottom end of the hanger was connected to the T-plate by bolts. At the bottom of the bionic mechanical foot test platform, a rectangular sand trough was placed, which was covered with white quartz sand with a thickness of about 600 mm. Two high-speed cameras were placed on the same side of the test platform (Figure 4). Before testing, the orientations of two high-speed cameras were adjusted to ensure that the movement picture of the bionic mechanical foot appeared in the screen of cameras at the same time.

**Figure 5:**
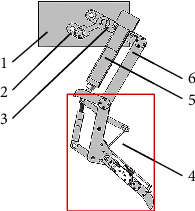
Mechanical leg for testing the bionic mechanical foot: (1) T-plate, (2) motor transmission shaft, (3) imitated femur structure, (4) bionic mechanical foot, (5) actuator of the foot, and (6) imitated tibia structure.

In order to test the performance of the bionic mechanical foot, we designed a mechanical leg that imitated the structure of the ostrich hindlimb (Figure 5). Its transmission part adopted a crank rocker mechanism, and the motor that controls the locomotion of the leg was installed on T-plate. The T-plate was a key component that connected the mechanical leg to the test platform, allowing the mechanical leg to move only within the sagittal plane. The bionic mechanical foot was connected to test platform by the mechanical leg and can only move in the sagittal plane.

**Figure 6:**
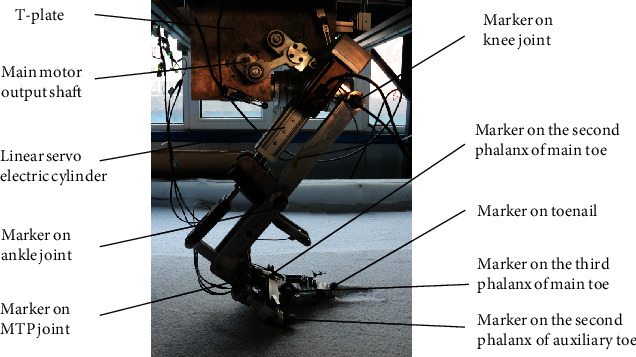
Position of the bionic mechanical foot markers.

In order to clearly record the trajectory of each marker, white reflective balls were pasted on the corresponding marker before the travelling performance test of the bionic mechanical foot on sand, and a total of 8 markers were selected (Figure 6). Except for the ankle joint of the bionic mechanical foot, the MTP joint, the first phalanx of the main toe, the second phalanx of the main toe, the third phalanx of the main toe, the toenail, and the second phalanx of the auxiliary toe, the knee joint of the mechanical leg was also considered a reference point. The fourth phalanx of the main toe and the toenail were connected by bolts, so trajectory of the toenail also represents trajectory of the fourth phalanx of the main toe.

### 3.2. Movement Parameter of the Bionic Mechanical Foot

The speed of the bionic mechanical foot was controlled by the main motor on the leg and linear servoelectric cylinder on the foot. The main motor controlled the forward speed of the foot, while the linear servoelectric cylinder controlled the lifting and falling speed of the foot. These two actuators cooperated to control the locomotion of the bionic mechanical foot. We set the low, medium, and high speeds to test the kinematics of the bionic mechanical foot on the loose sand and solid ground by changing the parameters of the main motor and linear servoelectric cylinder (Table 2). These six different working conditions were, respectively, named as solid ground low-speed locomotion, solid ground medium-speed locomotion, solid ground high-speed locomotion, loose sand low-speed locomotion, loose sand medium-speed locomotion, and loose sand high-speed locomotion (hereinafter referred to as S-L, S-M, S-H, L-L, L-M, and L-H, respectively).

Before the test, the space calibration was carried out on the loose sand and the solid ground, respectively, by using the calibration frame. The sand was leveled before each test, and the parallelism of sand was measured with the infrared tester to ensure that the angle displayed did not exceed 0.1°. A hardwood board was laid flat on sand as the solid ground, and the solid ground was consistent with the height of the loose sand. The size of the hardwood board was 2400 mm × 350 mm × 30 mm. The bionic mechanical foot moved from the same initial position in the same posture at the beginning of each test. Moreover, the vertical load of the bionic mechanical foot was constant when it was tested on solid ground and the loose sand. Three repeated tests were carried out for each working condition and the results were averaged.

### 3.3. Plantar Pressure Test and Footprint Acquisition

The plantar pressure acquisition of the bionic mechanical foot was carried out by the piezoresistive flexible film pressure sensor (D2027). The plantar pressure was calculated according to the weight of the bionic mechanical foot and mechanical leg, as well as effective area of the foot. Finally, a thin-film pressure sensor with a measuring range of 15-25 kg was selected. The outer diameter of the sensor was 27 mm, and the diameter of the sensing area was 20 mm. According to the structure of the bionic mechanical foot and data collection requirements, we attached three thin-film pressure sensors to the bottom of the third phalanx of the main toe, the second phalanx of the auxiliary toe, and the toenail.

**Figure 7:**
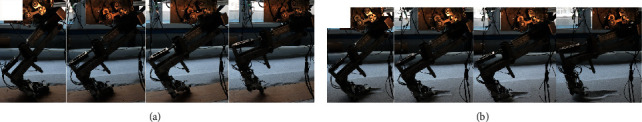
The travelling process of the bionic mechanical foot. (a) Travelling process on solid ground. (b) Travelling process on loose sand.

The test was also carried out under six working conditions; the movement speed of the bionic mechanical foot was consistent with that in the movement parameter collection (Figure 7). The bionic mechanical foot had obvious disturbance to the sand when it moved on the loose san, and formed a sinkage footprint. There were significant differences in footprints at different movement speeds.

## 4. Result and Discussion

### 4.1. Analyses in Movement Parameters

The movement video of the bionic mechanical foot was processed by Simi Motion, and the kinematic data of each marker was extracted. A total of 12 videos were processed in this test, with 6 for the solid ground and 6 for the loose sand. The average speed and average foot spacing of the bionic mechanical foot under six working conditions are shown in Table 3.

On the same ground, the average speed and average foot spacing of the bionic mechanical foot gradually increased with increase of the running speed. When the main motor and linear servoelectric cylinder were set to operate at medium and low speeds, the average speed of the bionic mechanical foot on the loose sand was higher than that on the solid ground. When the main motor and linear servoelectric cylinder were set to operate at high speed, the average speed of the bionic mechanical foot on the solid ground was higher than that on the loose sand, and the average foot spacing was also significantly larger. It is difficult to see the performance difference between the bionic mechanical foot on the solid ground and loose sand from the average speed, so it is necessary to further analyze the kinematics performance of the bionic mechanical foot.

**Figure 8:**
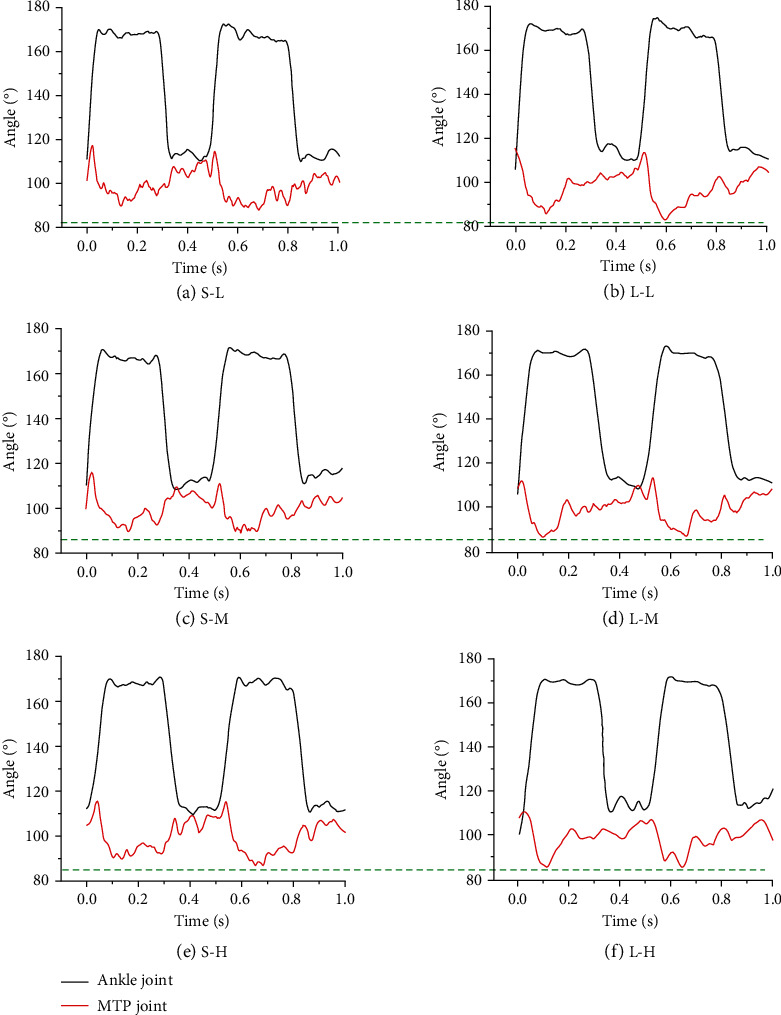
The angle changes curve of the ankle joint and the MTP joint. (a–f) The angle changes of each joint under six working conditions.

The angle changes curve of the ankle joint and the MTP joint of the bionic mechanical foot is shown in Figure 8. When the bionic mechanical foot moved on the solid ground, the speed had no significant influence on the angle changes of the ankle joint. The angle of the ankle joint increased first and then decreased, with a variation range of 110°~170°. When the bionic mechanical foot moved on a different ground, the angle of the MTP joint first decreased and then increased, with a variation range of 75°~115°. When running at high speed, the amplitude of the angle changes the curve of the ankle joint of the bionic mechanical foot on the loose sand was larger than that on the solid ground, and the ankle joint can reach a lower angle. The time for the MTP joint to reach the minimum angle was shorter and shorter with the increase of speed, and the angle change curve of the MTP joint was relatively gentle when moving on loose sand, which indicated that the bionic mechanical foot has better smoothness on the loose sand. When the bionic mechanical foot moved on the loose sand, the angle fluctuation of the ankle joint was larger at high speed than at low and medium speed, indicating that the bionic mechanical foot may be more suitable for moving at low speed and medium speed.

**Figure 9:**
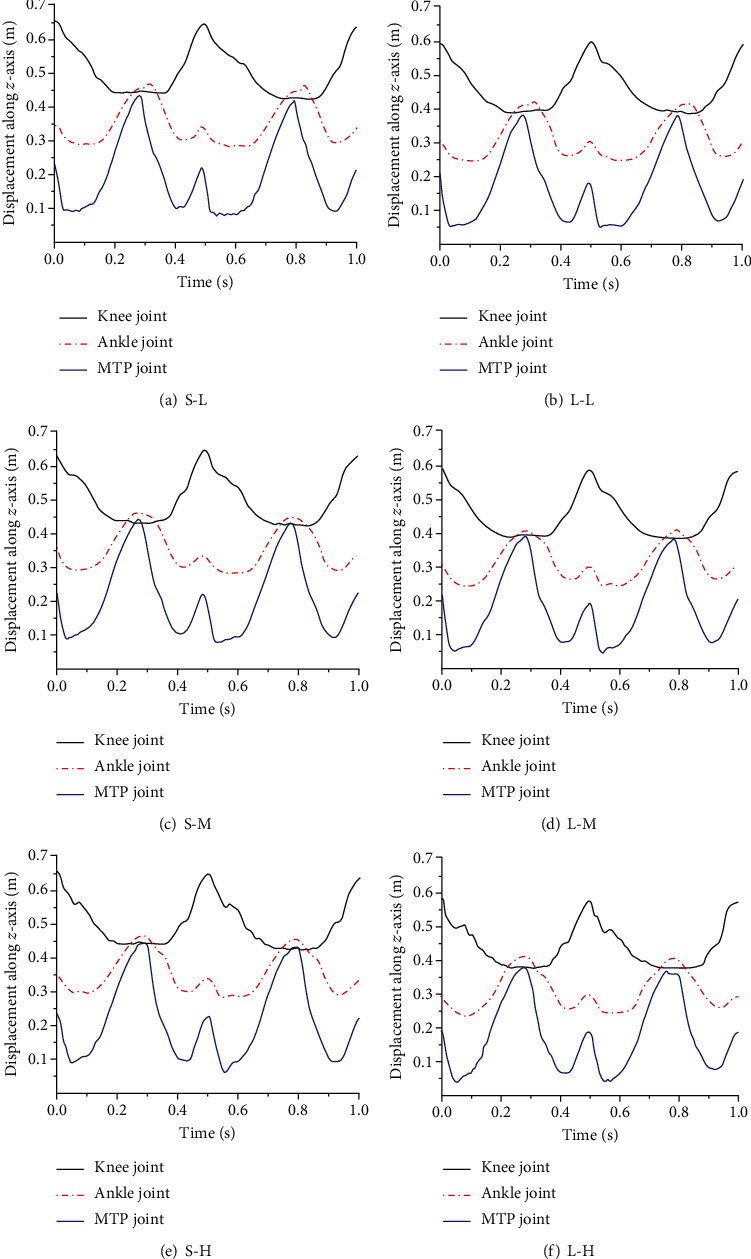
The displacement curve of the knee joint, ankle joint, and MTP joint along the *z*-axis. (a–f) The displacement changes of each joint along the *z*-axis under six working conditions.

The displacement curve of the ankle joint, the MTP joint of the bionic mechanical foot, and the knee joint along the *z*-axis is shown in Figure 9. At the same speed, the displacement curve of the knee joint, ankle joint, and MTP joint along the *z*-axis when the bionic mechanical foot moved on the loose sand was gentler than that on the solid ground. This indicated that the elastic element can play a better role in energy storage and shock absorption, making the bionic mechanical foot more stable when moving on the loose sand. Since there was some error in the calibration height between the loose sand and the solid ground, the initial height of displacement of each joint on the solid ground along the *z*-axis was different from that on the loose sand. However, no matter on the solid ground or loose sand, the MTP joint still had a certain distance from the ground at the lowest point. This indicated that the MTP joint was not in contact with the ground during locomotion, and it can achieve the function of leaving the ground permanently.

**Figure 10:**
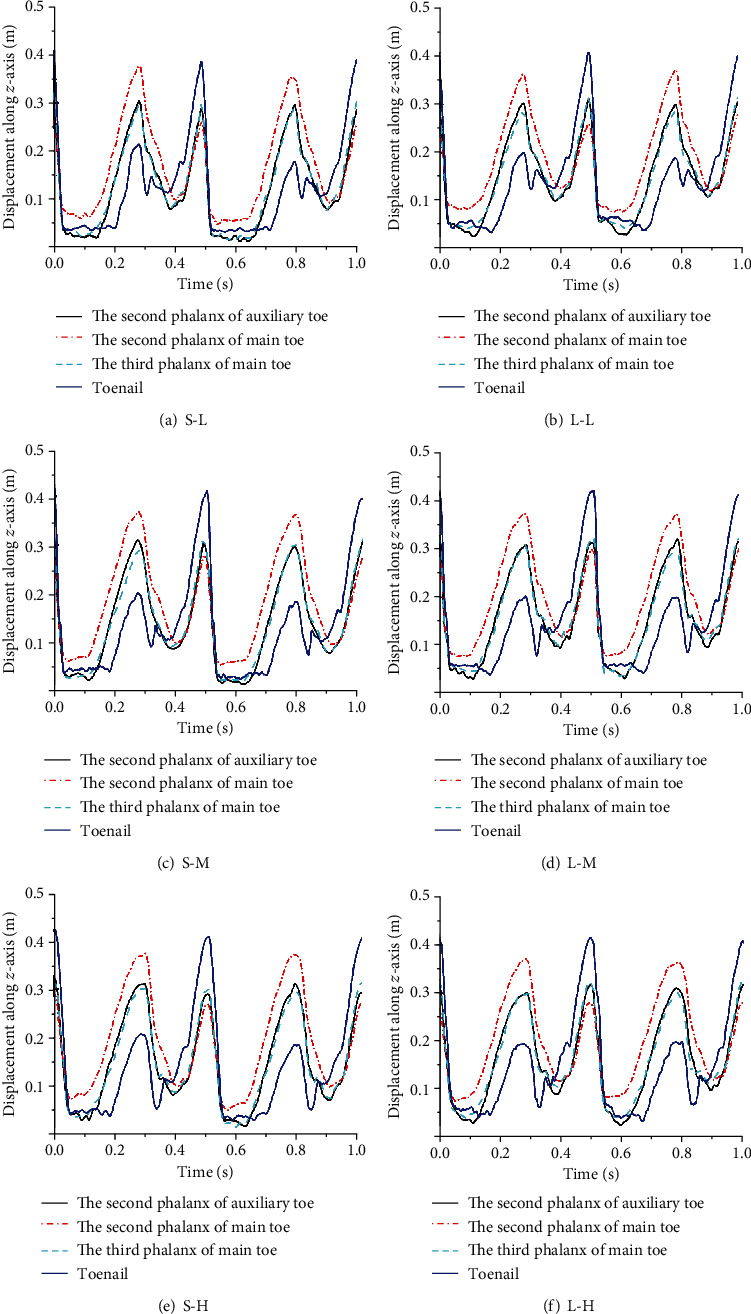
The displacement curve of the phalanges along the *z*-axis. (a–f) The displacement changes of each phalanx along the *z*-axis under six working conditions.

In order to study the movement rule of the phalanges of the bionic mechanical foot, the second phalanx of the auxiliary toe, the second phalanx of the main toe, the third phalanx of the main toe, and the toenail were selected as the research objects. The displacement curve of the phalanges of the bionic mechanical foot along the *z*-axis is shown in Figure 10. The figure shows that the phalanges of the bionic mechanical foot touched the ground at the same time, and the second phalanx of the main toe, the third phalanx of the main toe, and the toenail left the ground in order (The phalanges were in direct contact with the ground when the displacements of phalanges along the *z*-axis approached 0.). The higher the speed, the shorter the contact time between the phalanges and the ground when moving on the same ground. At the same speed, the contact time between each phalanx and the solid ground was significantly higher than that of the loose sand, indicating that the bionic mechanical foot has better adaptability on the loose sand.

When the bionic mechanical foot moved at medium and low speeds, its movement speed on the loose sand was higher than that on the solid ground, while the situation was just the opposite when moving at high speed. Through the analyses of the angle changes of the ankle joint and MTP joint, as well as the displacement changes of the knee joint, ankle joint, MTP joint, and phalanges along the *z*-axis, it can be seen that the locomotion of the bionic mechanical foot on the loose sand was more smoother than that on the solid ground. The main reason was that the energy storage and shock absorption mechanism, the flexible passive control mechanism, and the elastic elements at each phalangeal joint make the bionic mechanical foot have good flexibility and reduce the impact force from the ground. Furthermore, each phalanx of the bionic mechanical foot can touch the ground at the same time and leave the ground in order. The toenail finally left the ground and thrust against the ground, providing the main traction for leg. Therefore, it can be concluded that the excellent structural characteristics of the bionic mechanical foot make it have a good adaptability on the loose sand.

### 4.2. Analyses in Plantar Pressure and Footprint

**Figure 11:**
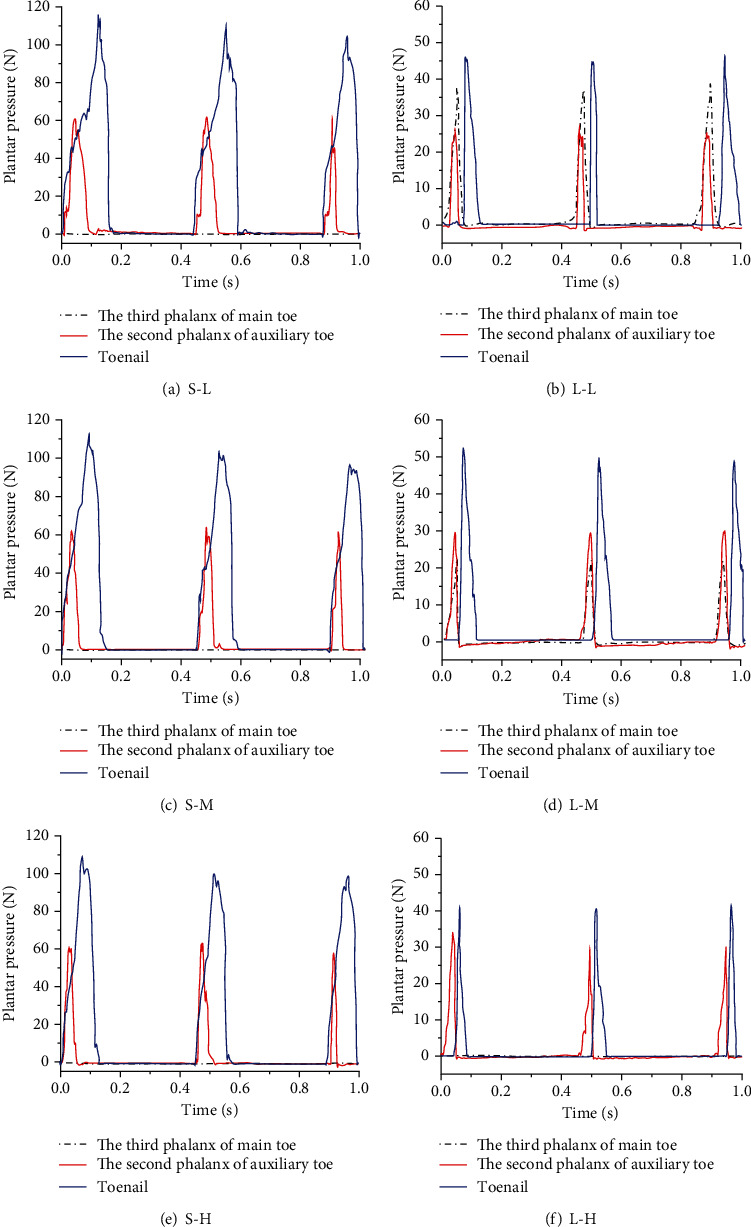
The plantar pressure change curve of the bionic mechanical foot. (a–f) The plantar pressure changes under six working conditions.

The curve of the plantar pressure of the bionic mechanical foot under six working conditions is shown in Figure 11. The results of the plantar pressure test on the solid ground showed that only the toenail and the second phalanx of the auxiliary toe were in contact with ground during the locomotion of the bionic mechanical foot. The toenail and the second phalanx of the auxiliary toe touched the ground at the same time, and the second phalanx of the auxiliary toe left ground first relative to the toenail. The results of the plantar pressure test on the loose sand showed that when the bionic mechanical foot moved at a low speed on the loose sand, the third phalanx of the main toe and the second phalanx of the auxiliary toe touched the ground at the same time. This indicated that the middle part of the bionic mechanical foot first touched the ground, and then completely touched ground. The order of leaving the ground was the second phalanx of the auxiliary toe, the third phalanx of the main toe, and the toenail. When the bionic mechanical foot moved at medium speed on loose sand, compared with the low-speed locomotion, the pressure on the third phalanx of the main toe decreased, while the pressure on the second phalanx and toenail of the auxiliary toe increased. When the bionic mechanical foot moved at high speed on loose sand, the third phalanx of the main toe was not under pressure. Therefore, the third phalanx of the main toe in contact with the ground when the bionic mechanical foot moved at medium and low speeds and did not get in contact with the ground at high speed.

When the bionic mechanical foot moved on the solid ground, the middle part of its pelma had no direct contact with ground, and the traction was mainly provided by the front end of the main toe and the auxiliary toe. When the bionic mechanical foot moved at medium and low speeds on the loose sand, its movement posture was similar to the real ostrich foot. When it touched the ground, each phalanx contacted the ground at the same time, and when it left the ground, each phalanx left the ground in order. Moreover, the plantar pressure of the bionic mechanical foot on the solid ground was higher than that on the loose sand, and the contact time between the bionic mechanical foot and the ground was longer than that on the loose sand. Through the analyses of the change of plantar pressure, the bionic mechanical foot designed in this paper is more suitable for medium-speed and low-speed locomotion on the loose sand; it is helpful to reduce the sinkage and improve the trafficability on the loose sand.

**Figure 12:**
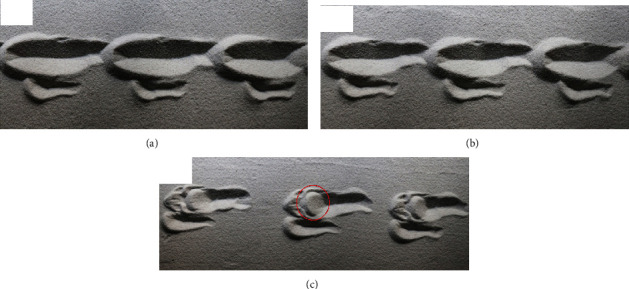
The footprints of the bionic mechanical foot on the loose sand. (a) Footprints at low speed. (b) Footprints at medium speed. (c) Footprints at high speed.

The footprints of the bionic mechanical foot on the loose sand at different speeds were shown in Figure 12. The contour of footprints left by the bionic mechanical foot on the loose sand at medium speed was similar to that at low speed, but the footprint depth at medium speed was slightly shallower than that at low speed. When high-speed locomotion, the time that toenail touched ground was short, so the footprint contour at high speed was quite different from medium-speed and low-speed locomotion. When the bionic mechanical foot moved on the loose sand, with the increase of speed, although the footprint depth of the toenail did not change much, the plantar footprint depth and disturbance range on the sand were getting smaller and smaller. It showed that the bionic mechanical foot has good antisinkage ability on loose sand, which is helpful to improve its trafficability on loose sand.

## 5. Conclusion

In this paper, a bionic mechanical foot suitable for locomotion on loose sand was designed based on the structural characteristics of the ostrich foot and its rigid-flexible coupling mechanism of the tendon-bone synergies. Like the real ostrich foot, the mechanical foot can open and close its two toe structure when moving, with the phalanges of the main toe successively off the ground, and the toenail thrust against the ground. The travelling performance of the bionic mechanical foot on the loose sand was tested on a test platform by using Simi Motion.

The speed of the bionic mechanical foot under six working conditions (S-L, S-M, S-H, L-L, L-M, and L-H) was, respectively, 27.22 mm/s, 42.60 mm/s, 64.00 mm/s, 28.31 mm/s, 46.92 mm/s, and 60.43 mm/s. When the bionic mechanical foot moved at medium and low speeds, its movement speed on the loose sand was higher than that on the solid ground, while the situation was just the opposite when moving at high speed. By analyzing the angle changes of the ankle joint and MTP joint and the displacement changes of the knee joint, ankle joint, MTP joint, and each phalanx along the *z*-axis during the locomotion of the bionic mechanical foot, compared with solid ground, the bionic mechanical foot has a good adaptability on the loose sand. The results of the plantar pressure test showed that when the bionic mechanical foot moved on the solid ground, the second phalanx of the main toe did not touch the ground, and the pressure of the toenail and the second phalanx of the auxiliary toe was about twice than that on the loose sand. When the bionic mechanical foot moved on the loose sand, the footprint depth and disturbance range of the foot became smaller and smaller with increasing speed. Therefore, the bionic mechanical foot has excellent travelling performance on sand and can realize the movement form that each phalanx touches the ground at the same time and leaves the ground in order, just like the postures of ostrich foot. The toenail thrust against the ground when the bionic mechanical foot left the ground, which played a role in improving the traction.

## Figures and Tables

**Table 1:** Specifications of the bionic mechanical foot.

Name	Value
The main toe (mm)	320
The auxiliary toe (mm)	165
Imitated ligament structure (mm)	120
Weight (kg)	8.2

**Table 2:** Speed parameters of the actuator on the bionic mechanical foot.

Name	Low speed	Medium speed	High speed
Main motor frequency	6.00	8.00	10.00
Linear servoelectric cylinder frequency	38000	42000	46000
Linear servoelectric cylinder pulse	46500	46500	46500

**Table 3:** Average speed and average foot spacing of the bionic mechanical foot.

Working conditions	Average speed (mm/s)	Average foot spacing (mm)
S-L	27.22	506.0
S-M	42.60	554.5
S-H	64.00	639.5
L-L	28.31	505.0
L-M	46.92	564.0
L-H	60.43	591.0

## Data Availability

The data used to support the findings of this study are available from the corresponding author upon request.
